# The development of cognitive control in children with chromosome 22q11.2 deletion syndrome

**DOI:** 10.3389/fpsyg.2014.00566

**Published:** 2014-06-10

**Authors:** Heather M. Shapiro, Flora Tassone, Nimrah S. Choudhary, Tony J. Simon

**Affiliations:** ^1^Department of Psychiatry and Behavioral Sciences, MIND Institute, University of California at DavisSacramento, CA, USA; ^2^Department of Biochemistry and Molecular Medicine, University of California at DavisSacramento, CA, USA

**Keywords:** 22q11.2 deletion syndrome, cognitive control, executive function, childhood cognitive development, developmental disorders, catechol-O-methyltransferase (COMT)

## Abstract

Chromosome 22q11.2 Deletion Syndrome (22q11.2DS) is caused by the most common human microdeletion, and it is associated with cognitive impairments across many domains. While impairments in cognitive control have been described in children with 22q11.2DS, the nature and development of these impairments are not clear. Children with 22q11.2DS and typically developing children (TD) were tested on four well-validated tasks aimed at measuring specific foundational components of cognitive control: response inhibition, cognitive flexibility, and working memory. Molecular assays were also conducted in order to examine genotype of catechol-O-methyltransferase (COMT), a gene located within the deleted region in 22q11.2DS and hypothesized to play a role in cognitive control. Mixed model regression analyses were used to examine group differences, as well as age-related effects on cognitive control component processes in a cross-sectional analysis. Regression models with COMT genotype were also conducted in order to examine potential effects of the different variants of the gene. Response inhibition, cognitive flexibility, and working memory were impaired in children with 22q11.2DS relative to TD children, even after accounting for global intellectual functioning (as measured by full-scale IQ). When compared with TD individuals, children with 22q11.2DS demonstrated atypical age-related patterns of response inhibition and cognitive flexibility. Both groups demonstrated typical age-related associations with working memory. The results of this cross-sectional analysis suggest a specific aberration in the development of systems mediating response inhibition in a sub-set of children with 22q11.2DS. It will be important to follow up with longitudinal analyses to directly examine these developmental trajectories, and correlate neurocognitive variables with clinical and adaptive outcome measures.

## Introduction

Chromosome 22q11.2 Deletion Syndrome (22q11.2DS) results from a 1.5- to 3-megabase microdeletion on the long (q) arm of chromosome 22 (Carlson et al., 1997) and occurs in approximately one in 2000–4000 live births (Oskarsdóttir et al., 2004; Shprintzen, 2008). Children with this disorder have mild to moderate intellectual impairments (median full scale IQ 70 ± 15) (Scambler, 2000) and a cognitive profile with difficulties on a range of functions including attention and quantitative processing (Simon et al., 2005; Simon, 2008; Simon and Luck, 2011), as well as cognitive control (Bish et al., 2005; Sobin et al., 2005). Importantly, children with 22q11.2DS also have behavioral impairments and are at significantly increased risk for developing schizophrenia in adulthood (Murphy et al., 1999). Approximately 25% of individuals with 22q11.2DS will develop schizophrenia by adulthood (Bassett et al., 2003), rendering it the highest genetic risk factor for the disorder after having a monozygotic twin or two parents with schizophrenia.

In the schizophrenia literature, impairments in cognitive control have been shown to precede symptom onset (Cannon et al., 2003; Brewer et al., 2005; Lencz et al., 2006). There is also evidence for attenuated cognitive control impairments among first-degree relatives of individuals with schizophrenia, suggesting that these deficits might be part of an endophenotype related to genetic susceptibility for the disorder (Snitz et al., 2006). Based on this line of evidence, a better understanding of cognitive control component processes in children with 22q11.2DS, a group with a genetically conferred risk for schizophrenia, might help to identify specific cognitive functions that could act both as biomarkers for conversion risk, and as specific targets for intervention that might reduce that risk.

In the current study, our goal was to take a first step toward characterizing the nature and extent of cognitive control impairments throughout development in children with 22q11.2DS by conducting a cross-sectional analysis in individuals aged 7–14 years. Cognitive control, a term largely synonymous with executive function, describes the dynamic system of mental processes that directs and regulates cognitive resources in order to maximally achieve one's goals. Miyake et al. (2000) described a theoretical framework suggesting that this system encompasses three foundational cognitive control components, namely response inhibition, cognitive flexibility, and working memory, and that these components are both distinct and interrelated. This system is not static developmentally, but rather each component process has a unique developmental trajectory, and the degree to which the components are distinct or interrelated changes as a function of age (Best and Miller, 2010).

Preliminary evidence suggests that children with 22q11.2DS exhibit impairments in cognitive control processes, as well as neuroanatomical and neurofunctional aberrations in networks believed to support cognitive control processes. In a study aimed at understanding schizophrenia-like cognitive impairments in children with 22q11.2DS aged 7–16 years, Lewandowski et al. (2007) found that performance on a Wisconsin Card Sort task, a well-established paradigm for examining cognitive flexibility, was impaired relative to TD, even after controlling for general intellectual function by including IQ as a regressor in the statistical models. By contrast, working memory impairments, as measured by the Children's California Verbal Learning Test (CVLT-C), were not significant after accounting for IQ in the regression models.

Campbell et al. (2010) also tested cognitive control abilities in children with 22q11.2DS, aged 6–16 years. They found that children with 22q11.2DS had significantly impaired cognitive flexibility relative to TD, as measured by the Switch task from the Maudsley Attention and Response Suppression battery, as well as impaired working memory, as measured by the Children's Memory Scale and a Spatial Working Memory task from the Cambridge Neuropsychological Testing Automated Battery. By contrast, they found no between-group differences on a Go/No-Go task, a well-established paradigm for examining response inhibition. Other studies, however, demonstrated that children with 22q11.2DS had inhibitory control impairments on tasks requiring interference control (Bish et al., 2005) and oculomotor inhibition (Sobin et al., 2005).

Thus, it is evident that while cognitive control systems appear to be impaired in 22q11.2DS, the specific nature of these impairments is unclear. There are a number of factors that could account for differences in the previous literature. First of all, some of the cognitive control measures were extracted from psychometrically well-characterized, standardized behavioral testing instruments. While these tests are valuable, they are not as good at isolating specific cognitive processes, as are experimental neurocognitive tests. Additionally, the previous studies characterized large age ranges, throughout which cognitive control processes are dynamically changing as a function of brain developmental processes. Thus, given the relevance of these impairments to cognitive function in 22q11.2DS, as well as to schizophrenia risk, it is important to characterize the nature and developmental trajectory of cognitive control processes using most sensitive, specific neurocognitive tests of cognitive control component processes.

Preliminary evidence from a cross-sectional sample of individuals aged 7–14 years reported that children with 22q11.2DS had an age-related impairment in the executive control of attention, specifically with respect to a flanker inhibition paradigm (Stoddard et al., 2011). Interestingly, another cross-sectional study examining a complementary aspect of attention, namely attentional orienting, in the same age range demonstrated the opposite pattern: performance in older individuals with 22q11.2DS was significantly better and less variable than that of their younger counterparts (Shapiro et al., 2012). This pattern suggests that different systems of attention and their underlying neural networks are developing with different trajectories in 22q11.2DS. Importantly, it appears that impairments in cognitive control, not general cognitive or attentional function, are preceding the risk period, and might contribute to part of a risk profile. Testing this hypothesis is important for understanding networks that might be particularly plastic in a critical age period during which aberrant neurodevelopment might render a subset of individuals at increased risk for developing schizophrenia.

Here we tested an age range of children with 22q11.2DS and TD comparison children aged 7–14 years on a battery of specific cognitive control component processes for a cross-sectional analysis of the development of cognitive control in this population. Based on Miyake et al.'s (2000) theoretical model of cognitive control foundational components, we examined response inhibition, cognitive flexibility, and working memory using a battery of child-adapted, well-validated neurocognitive tasks to probe each component. Response inhibition was assessed with a canonical stroop task (Stroop, 1935). The second task for measuring response inhibition was a child-friendly “whack-a-mole” version of a Go/No-Go task. Go/No-Go tasks have been widely used in both typically and atypically developing children to examine inhibitory control (Casey et al., 1997). Here participants responded to a frequently occurring target (“Go” trial), and inhibited the pre-potent response to an infrequent target (“No-Go” trial). Cognitive flexibility was examined using a Visually-Cued Card Sort (VCCS), a downward extension of the Wisconsin Card Sort that is geared toward children (Zelazo et al., 2004). In this study participants sorted cards according to rules about shape or color, and the sorting rules changed according to certain criteria. In contrast to the Wisconsin Card Sort, participants received an explicit visual cue indicating the specific rule set by which to sort. Finally a Self-Ordered Pointing Test (SOPT) was used to examine working memory (Petrides and Milner, 1982). Participants identified and responded to a sequence of images, remembering which images they have previously chosen, and select a new image on each subsequent trial.

Beyond age-related associations with cognitive control, we wanted to examine additional factors that might contribute to cognitive control performance in children with 22q11.2DS. The gene for catechol-O-methyltransferase (COMT) is located within the deleted region in 22q11.2DS and is an important regulator of prefrontal dopamine (DA), a neurotransmitter that has previously been reported to play a role in higher-level cognitive processes (Kimberg and D'Esposito, 2003). Given that children with 22q11.2DS have only a single copy of the COMT allele, it is likely that DA modulation is abnormal in these individuals. Importantly, the COMT gene contains two different allelic variations: Val and Met for high and low enzymatic activity, respectively. Previous studies of COMT genotype in 22q11.2DS have yielded differential results, with some studies reporting Met hemizygosity of COMT to be related to poorer outcome on tasks requiring executive control (Baker et al., 2005; Takarae et al., 2009), and others reporting better outcomes (Bearden et al., 2004; Shashi et al., 2006). Additional studies have found no relationship between COMT genotype and measures of cognitive control in 22q11.2DS (Glaser et al., 2006; Campbell et al., 2010). Thus, in order to investigate this relationship further, we examined cognitive control performance of the participants in this study as a function of COMT variant.

Based on previous evidence of cognitive control impairments in 22q11.2DS, we hypothesized that individuals with the disorder would perform more poorly on the cognitive control tasks relative to TD comparison children. Additionally, we hypothesized that a cross-sectional analysis of cognitive control development would reveal atypical developmental trajectories of specific cognitive control components, with worse performance in older but not younger children with 22q11.2DS, and that this pattern would be true in some but not all of the children.

## Materials and methods

### Participants

Seventy-one children with chromosome 22q11.2 deletion syndrome (mean age = 11.4[2.5] years; 31 female and 40 male) and 52 typically developing (TD) comparison children (mean age = 10.6[2.2] years; 27 female and 25 male), from 7 to 14 years of age, participated in the study. Data on IQ from the Wechsler Intelligence Scale for Children—4th edition (WISC-IV) (Wechsler, 2003) or the Wechsler Abbreviated Scale of Intelligence (WASI) (Wechsler, 1999) was available from a subset of participants: 55 children with 22q11.2DS and 38 TD participants. Full-scale IQ (FSIQ) ranged from 46 to 103 for children with 22q11.2DS and 80 to 154 for TD children. Biological samples were available for genotyping on 58 of the children with 22q11.2DS. Of these individuals, 31 were hemizygous for the COMT Val allele and 27 were hemizygous for the COMT Met allele. A subsample of the study participants (12 with 22q11.2DS and 8 TD) performed the cognitive task battery at a conference where they did not complete the WASI or submit biological samples, thus contributing to incomplete IQ and COMT data, respectively. Exclusion criteria for both groups included head injury or other focal neurological abnormality. Exclusion criteria for TD participants were the presence of any other learning or behavioral/psychiatric disorder. Additional exclusion criteria on an individual task basis are described under the description for each task below. One participant with 22q11.2DS met exclusion criteria for all tasks and was removed from analysis, resulting in the final sample of 71 children with 22q11.2DS and 52 TD children that are described here. The parents of all participants provided written informed consent based on protocols approved by the Institutional Review Board at the University of California, Davis. Table 1 depicts the demographic information for children in each group.

**Table 1:** **Demographic data on children with 22q11.2DS (22q) and TD children**.

**Cognitive task**		***N***	**Age in years: Mean (*SD*)**	**FSIQ: Mean (*SD*)**	**Gender: Sample size (Male/Female)**	**COMT: Sample size (Val/Met)**
Stroop	22q	39	11.4 (2.5)	74.6 (14.2)	21/18	21/14
	TD	29	10.5 (2.1)	114.0 (12.1)	17/12	NA
Go/No-Go	22q	64	11.5 (2.6)	73.9 (12.7)	37/27	28/25
	TD	49	10.7 (2.2)	113.2 (13.7)	24/25	NA
VCCS	22q	62	11.6 (2.5)	73.7 (13.2)	34/28	28/21
	TD	50	10.7 (2.2)	113.2 (13.8)	24/26	NA
SOPT (Verbal)	22q	65	11.5 (2.6)	73.8 (13.4)	36/28	28/24
	TD	52	10.6 (2.2)	112.7 (13.6)	25/27	NA
SOPT (Non-verbal)	22q	56	11.6 (2.6)	73.0 (11.9)	32/24	25/19
	TD	47	10.8 (2.2)	112.5 (13.4)	23/24	NA

### Molecular analyses

Genomic DNA was isolated from 3 ml of peripheral blood leukocytes using standard procedure (Qiagen, Valencia, CA). Genotyping analysis for the COMT Val^108/158^ Met was carried out by TaqMan SNP Genotyping Assay (rs4680; Applied Biosystems, Foster City, CA). PCR reaction contained COMT SNP genotyping assay mix, TaqMan master mix and 25 ng DNA per reaction. PCR conditions were 95°C for 10 min, followed by 40 Cycles of 92°C for 15 s and 60°C for 1 min. Allelic discrimination plate read was performed on an Applied Biosystems Real-Time PCR System using the Sequence Detection System (SDS) Software.

### Task procedure

All participants completed paradigms testing cognitive control component processes, including response inhibition, a cognitive flexibility, and working memory. Tasks were administered on the same Elo 1715L Desktop Touch monitor for all participants.

#### Response inhibition paradigms

**Figure 1:**
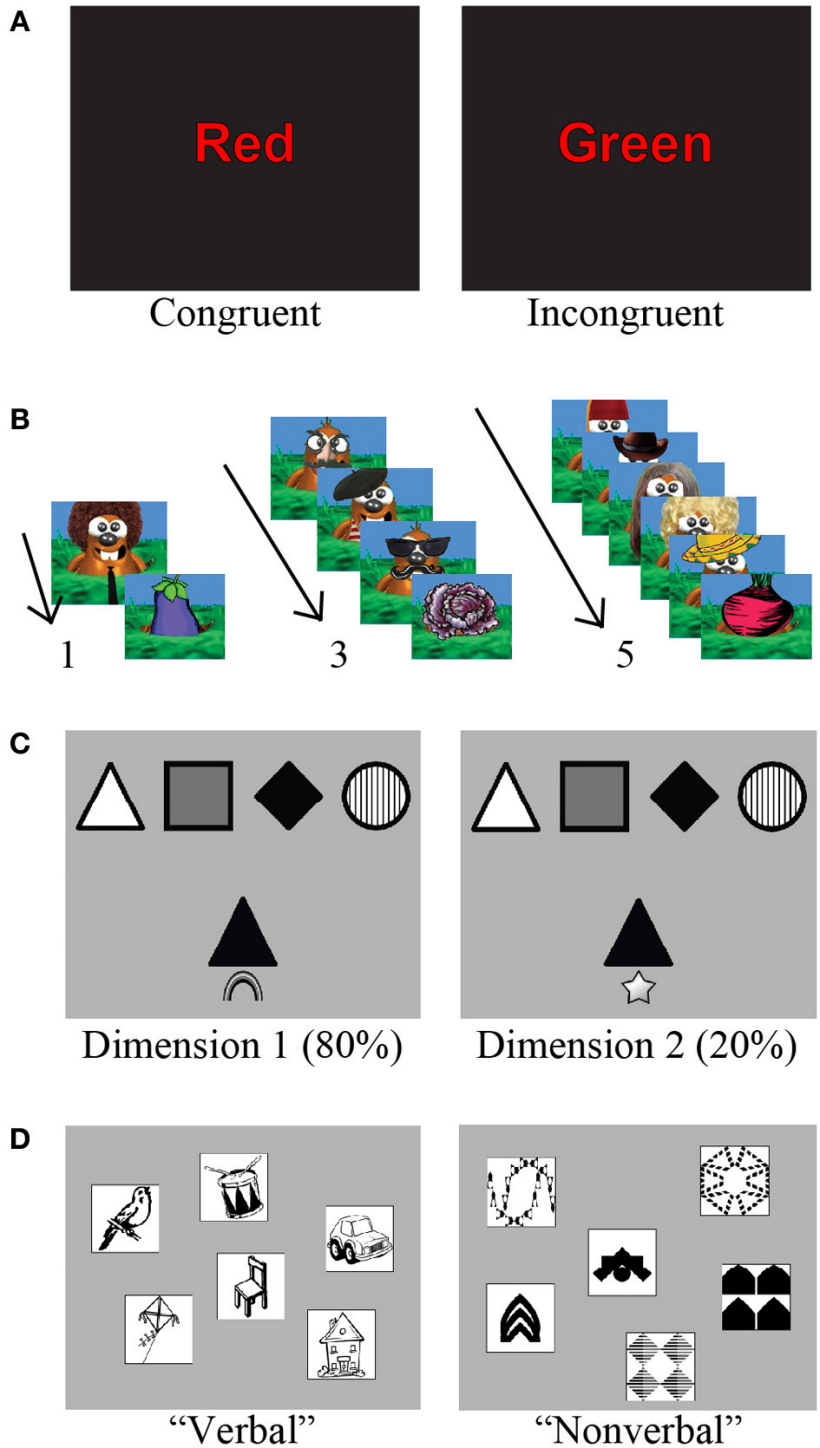
**Examples of the cognitive control battery. (A)** For the Stroop task, participants had to respond by indicating the ink color on congruent (left) or incongruent (right) trials. **(B)** “Whack-a-mole” Go/No-Go task. Children were instructed to press a button as quickly as possible when a cartoon mole appeared (Go trial), but to avoid pressing the button when a vegetable appeared (No-Go trial). No-Go trials were preceded by 1, 3, or 5 Go trials (Adapted from Shapiro et al., 2013). **(C)** The two gray squares each represent a touch-screen display in the Visually-Cued Card Sort (VCCS). A sample of the target cards can be seen at the top of the screen, while the test card is below. The visual cue appears just below the test card, with a rainbow indicating to sort by color (left panel), and a star indicating to sort by shape (right panel). **(D)** The two gray squares each represent a touch-screen display in the for the verbal and non-verbal Self-Ordered Pointing Test (SOPT), respectively. Images represent a trial with 6 objects, the most difficult condition of the task.

To examine response inhibition, participants completed a computerized version of the canonical Stroop task (Stroop, 1935). Participants were presented with stimuli on a monitor and asked to respond (by pressing one of three colored buttons) in which color font the stimulus was presented (red, green, or blue). In the congruent condition, participants were presented with the words “red,” “green,” or “blue” in the same font color as the presented word. In the incongruent condition, participants were presented with one of the same three color words; however, the word was presented in a font color that was *different* from the specified color word (Figure 1A). There were a total of 240 trials, with 168 and 72 congruent and incongruent trials, respectively. The rationale for this 70–30 congruent-incongruent ratio was to maintain the potency of the rule set for responding to the congruent color. Stimuli were presented for 2000 ms, or until the participant responded, with interstimulus intervals of 200, 500, or 750 ms. The dependent variable here was median response time (RT) on congruent relative to incongruent trials that were preceded by congruent vs. incongruent trials, respectively. Participants were excluded if they performed worse than chance (66.6% accuracy) on congruent or incongruent trials. Seven children with 22q11.2DS were excluded on this basis. This task was completed on a slightly smaller sample of participants (39 participants with 22q11.2DS and 29 TD), due to a modification of the task design that occurred approximately 6 months into the study.

Response inhibition was also measured using a child-adapted version of a Go/No-Go response inhibition task (Figure 1B). A subset of this data has been published previously (Shapiro et al., 2013), but our goal here was to extend those findings by including a larger sample of participants, and also examine within-subject differences on this component of the battery relative to the other cognitive control processes. For a full description of the task, please reference Shapiro et al. (2013). Key details are the task parameters including Go (75%) and No-Go (25%) trials. Stimuli were presented for 1000 ms, with interstimulus intervals of 200, 500, or 750 ms. Participants completed 20 trials of each No-Go type (preceded by one, three, or five Go trials, respectively), divided equally into four blocks. Primary outcome measures were accuracy and RT to Go and No-Go trials, respectively. Participants were excluded if they performed at lower than 75% accuracy when responding to the frequently occurring Go stimuli, or outside of 2 standard deviations from the mean for accuracy on No-Go trials. Seven children with 22q11.2DS and three TD participants were excluded on this basis.

#### Cognitive flexibility paradigm

To examine cognitive flexibility, participants completed a computerized version of the VCCS on a computer with a touch-screen monitor. This is a children's modified version of the Wisconsin card sorting task, and was adapted from a task by Zelazo et al. (2004) that proved to be effective at measuring perseverative behavior in a wide age range of children. At a distance of approximately 60 cm from the computer, participants viewed four target cards that displayed four different shapes (circle, square, diamond, triangle) in four different colors (black, white, gray, striped; Figure 1C). They were instructed to sort 50 test cards onto the appropriate target card. The test cards were presented one at a time at a central location beneath the row of target cards. The participants were instructed to sort their cards either by color or by shape, as indicated by the visual cue that appeared below their card. A rainbow was the visual cue that indicated to sort by color, while a star indicated to sort by shape. Forty out of 50 trials were cued to sort by one of the dimensions (color or shape), while the remaining 10 trials were cued to sort by the secondary dimension. For the first 45 participants (23 with 22q11.2DS and 22 TD), color was the primary dimension (Dimension 1), while the remaining 67 participants (39 with 22q11.2DS and 28 TD) were presented with shape as the primary dimension. The trials were uniformly randomized, such that one trial of the secondary sorting dimension (Dimension 2) appeared within every five trials. The participants completed a demonstration of the task, followed by four practice trials, after which they began the 50 test trials. Test cards were presented on the screen for as long as the participants needed to make a response. If the response was incorrect, the test and target cards remained on the screen until the participants selected the correct target, after which the screen refreshed and a new test and target cards were presented. Primary outcome measures were percent accuracy of correctly sorted cards for each dimension (Dimensions 1 and 2 for 80 and 20% frequency, respectively), as well as the ratio of accuracy from Dimension 2 divided by Dimension 1. The ratio score was intended isolate the costing of switching dimensions (i.e., cognitive flexibility) from general card sorting ability on the task. Participants were excluded if their overall performance accuracy was less than 50%, if they did not appear to understand the task after repeating the instructions, or if they did not comply with the task instructions. Nine children with 22q11.2DS and two TD participants were excluded based on these criteria.

#### Working memory paradigm

Participants completed a modified version of the SOPT, originally designed by Petrides and Milner (1982). There were two versions of the task: verbal and non-verbal. The verbal version consisted of single-syllable, concretely nameable objects while the non-verbal version involved visual stimuli that were difficult to name or encode verbally. Visual stimuli were chosen from the Dover Clip Art Series, a library of images that is available copyright-free at doverpublications.com. A computer screen displayed an array of images presented on a touch-screen monitor. There were three levels to this task. From easiest to most difficult, the levels involved three, four, or six images, respectively. The most difficult level (six images) can be seen in Figure 1D. The participants were asked to point to an object (touch the object on the touch-screen monitor), with the condition that on each subsequent trial they must point to a different object. Each time the participants pointed to an object, the screen refreshed and the relative positions of the images were rearranged at random. Each block consisted of the same number of trials as different objects on the screen. There were four blocks at each level. Primary outcome measures were span (number of correct responses prior to the first error) and number of errors. Participants were excluded if their overall performance accuracy was at chance, if they did not appear to understand the task after repeating the instructions, or if they did not comply with the task instructions. Based on these criteria, six children with 22q11.2DS were excluded from analysis of the verbal version of the task. Fifteen children with 22q11.2DS and five TD children were excluded from analysis of the non-verbal version.

### Data analysis

Data were processed using scripts written by HS in MatLab (version 7.8) to generate outcome variables from raw data. Mixed model regression analyses were used to determine the effects of between-subject variables (diagnosis group, gender, and testing location) and task variables on primary outcome measures. Age was included as a regressor to examine developmental effects in a cross-sectional analysis. Additional models included full-scale IQ as a regressor in order to assess the relationship of general intellectual abilities with cognitive control function. Finally, COMT genotype was included as a regressor in order to examine the potential relationship of specific genetic variants to the cognitive control processes.

## Results

### Response inhibition—stroop task

Response inhibition was measured by accuracy and RT on two different Trial Types: congruent and incongruent. Children with 22q11.2DS had overall worse accuracy than TD children [*F*_(1, 65)_ = 12.12, *p* = 0.0009], and there was a trend toward a significant Group × Trial Type interaction, such that children with 22q11.2DS had a relatively worse accuracy on the incongruent relative to congruent trials [*F*_(1, 66)_ = 3.30, *p* = 0.07]. There was no overall group difference in RT [*F*_(1, 65)_ = 2.32, *p* = 0.13], nor a Group × Trial Type interaction in RT [*F*_(1, 66)_ = 0.44, *p* = 0.51].

**Figure 2:**
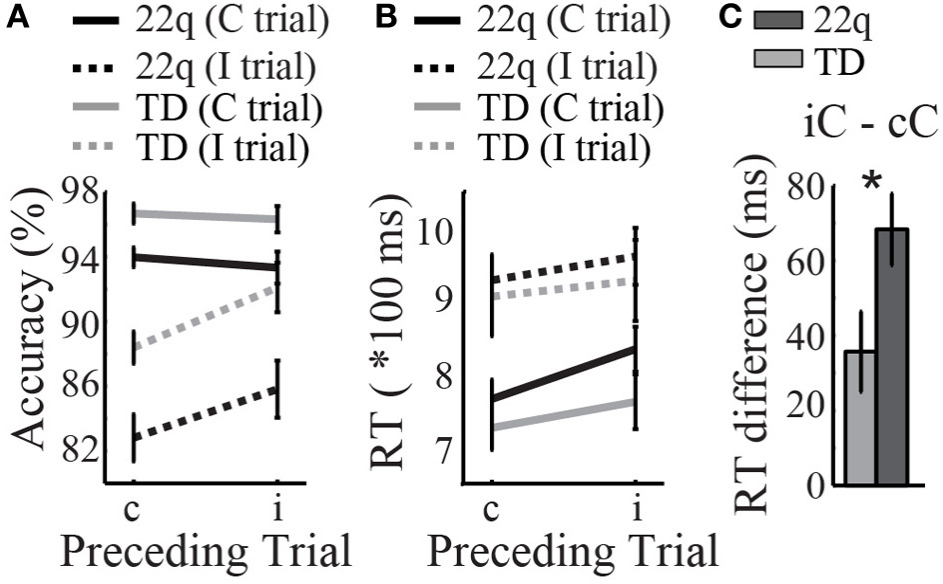
**Results of the response inhibition Stroop task. (A)** Children with 22q11.2DS had lower accuracy relative to TD participants across all trial types: Congruent (*C*) or Incongruent (*I*) trials preceded by congruent (*c*) or incongruent (*i*) trials, respectively. **(B)** Response time (RT) was similar between groups. **(C)** Children with 22q11.2DS were more greatly affected by a preceding interfering stimulus, as measured by a larger RT difference on congruent trials preceded by incongruent trials (*iC*) relative to congruent trials following other congruents trial (*cC*), ^*^p < 0.05.

In order to examine interference effects of preceding trial type, we next examined group performance as a function of four different Trial types: congruent and incongruent trials that were preceded by congruent or incongruent trials, respectively. Thus, the four different Trial Types included: congruent preceded by congruent (*cC*), congruent preceded by incongruent (*iC*), incongruent preceded by congruent (*cI*), and incongruent preceded by incongruent (*iI*). Within each Trial Type, children with 22q11.2DS had significantly worse accuracy relative to TD children across all Trial Types (Supplementary Table 1 and Figure 2A). By contrast, there were no group differences in RT for any of the specific trial types (Supplementary Table 1 and Figure 2B).

Next, we took the difference of RT on congruent trials that were preceded by incongruent trials (iC), minus that of congruent trials preceded by congruent trials (cC). The goal here was measure the specific interference effects of a prior incongruent trial on congruent RT, relative to RT on a congruent trial that is not preceded by an interfering stimulus (cC). Here we found that children with 22q11.2DS had a significantly larger RT difference (iC – cC) relative to that of TD children [*F*_(1, 65)_ = 5.06, *p* = 0.03], suggesting that this population is more greatly affected by the prior interfering stimulus (Supplementary Table 1 and Figure 2C).

### Response inhibition—Go/No-Go task

Performance on this task in children with 22q11.2DS has been previously reported (Shapiro et al., 2013) for a subgroup of children with 22q11.2DS (*n* = 47) and of TD children (*n* = 36). Here we report on the results of additional 17 children with 22q11.2DS and 13 TD children. These results are important to report here in order to compare individuals' performance across the additional cognitive control tasks.

**Figure 3:**
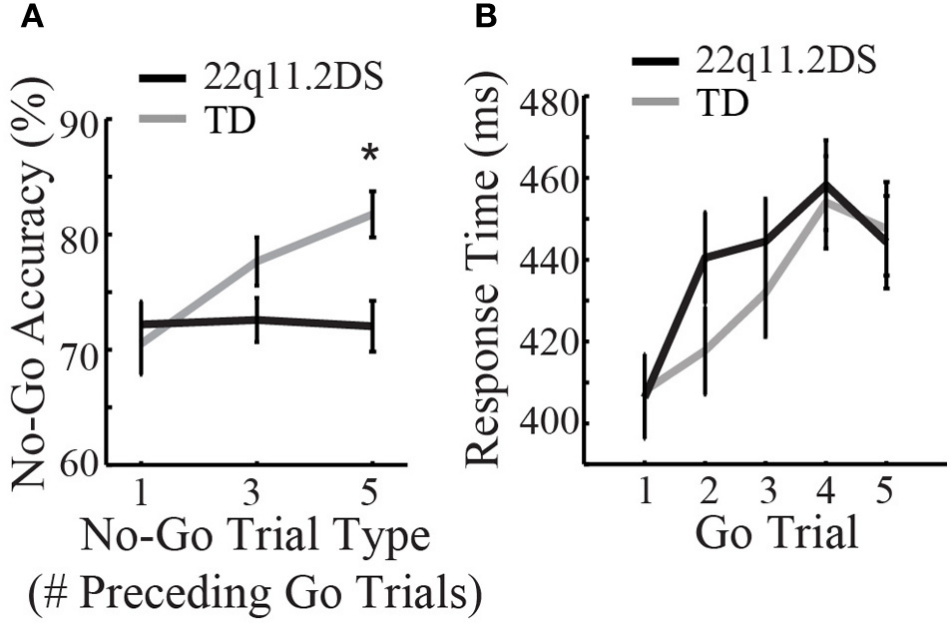
**Results of the response inhibition Go/No-Go task. (A)** No-Go accuracy differed between groups ^*^p < 0.05, while **(B)** response time on Go trials was similar between groups.

Response inhibition was measured by accuracy on No-Go trials that were parametrically manipulated for difficulty. The manipulation involved three different No-Go conditions, which included No-Go trials following one, three, or five Go trials, respectively. Diagnostic group, No-Go trial type, and gender were regressed on accuracy and RT. We found a significant Group × Trial Type interaction [*F*_(2, 222)_ = 6.54, *p* = 0.002; Figure 3A]. In order to understand this interaction better, we next examined the effects of No-Go condition within each group separately by regressing the No-Go condition on No-Go accuracy for each group. There was a significant effect of No-Go condition on accuracy in TD children, such that when No-Go trials were preceded by increasing numbers of Go trials, TD children had greater accuracy [*F*_(2, 96)_ = 11.51, *p* < 0.0001; mean accuracy = 70.5[18.7]%, 77.7[14.7]%, and 81.7[14.0]% for one, three, and five preceding Go trials, respectively]. By contrast, children with 22q11.2DS demonstrated no change in performance across conditions [*F*_(2, 126)_ = 0.036, *p* = 0.96; mean accuracy = 72.2[15.9]%, 72.6[15.3]%, and 72.0[17.6]% for one, three, and five preceding Go trials, respectively; Supplementary Table 2 and Figure 3A].

In order to examine if the group difference in response inhibition might be due to speed-accuracy trade-offs, RT was measured on consecutive Go trials leading up to a No-Go trial. Diagnostic group, gender, and Go trial number (one through five based on sequential order following a No-Go trial) were regressed on RT. There were no group differences in Go RT [*F*_(1, 110)_ = 0.22, *p* = 0.64; Supplementary Table 2 and Figure 3B]. Similarly, both groups demonstrated a similar performance pattern, consisting of a relative slowing from the first up to the fourth Go trial following a No-Go trial [*F*_(4, 192)_ = 30.1, *p* < 0.0001 for TD; *F*_(4, 252)_ = 31.2, *p* < 0.0001 for 22q11.2DS; Supplementary Table 2 and Figure 3B]. Thus, while response inhibition was impaired between groups (as measured by No-Go accuracy), this was not due to differences in RT on preceding Go trials.

### Cognitive flexibility—VCCS task

**Figure 4:**
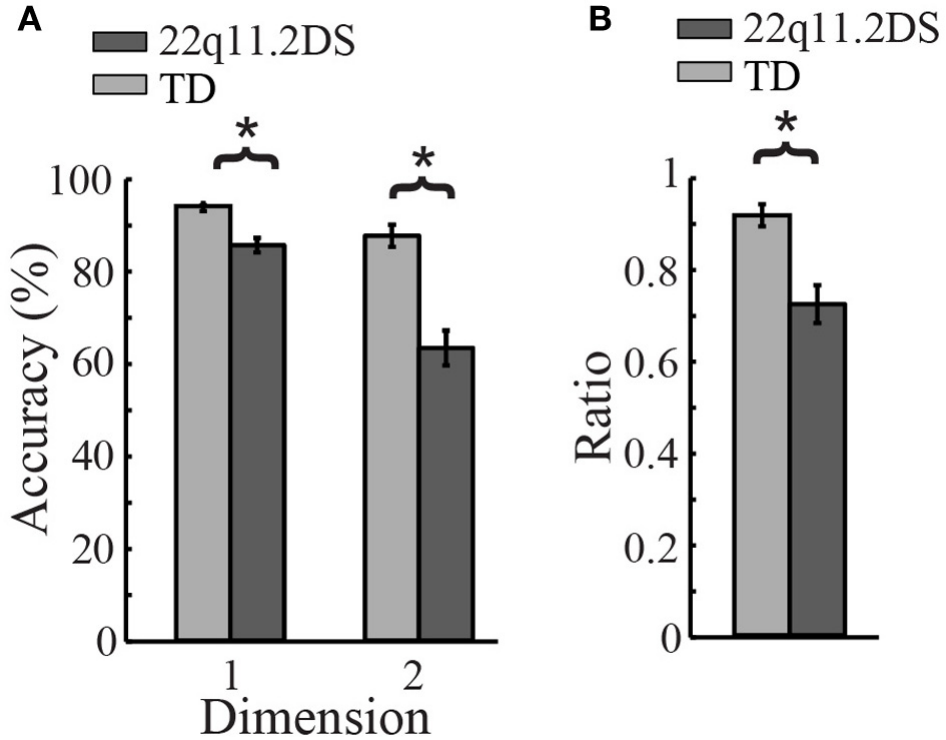
**Results of Visually-Cued Card Sort (VCCS), a test of cognitive flexibility. (A)** TD children had better accuracy when sorting by both dimensions (predominant and secondary, ^*^p < 0.05). **(B)** Children with 22q11.2DS performed significantly worse when sorting by the secondary dimension relative to the predominant dimension, as indicated by this group difference in the ratio score of accuracy on Dimension 2 divided by that of Dimension 1 (^*^p < 0.05).

To examine cognitive flexibility, percent accuracy was regressed against diagnostic group, sorting dimension (predominant or secondary), and gender. There was a significant group difference in accuracy [*F*_(1, 109)_ = 31.50, *p* < 0.0001; Figure 4A], as well as a significant Group × Dimension interaction, such that children with 22q11.2DS performed more poorly than TD children when sorting by the secondary dimension relative to the predominant dimension [*F*_(1, 110)_ = 13.41, *p* = 0.0004]. This was further supported by a significant group difference in the ratio score of accuracy on Dimension 2 divided by that of Dimension 1 [*F*_(1, 109)_ = 14.45, *p* = 0.0002; Figure 4B]. See Supplementary Table 3 for each group's percent accuracy on both dimensions, as well as the results of statistical tests for group differences in performance on each dimension.

### Working memory—SOPT task

There were two versions of this task (verbal and non-verbal), and each version had three levels of difficulty that from easiest to most difficult involved remembering three, four, or six images, respectively. To examine working memory performance, span and errors were regressed against diagnostic group, level of difficulty, and gender.

**Figure 5:**
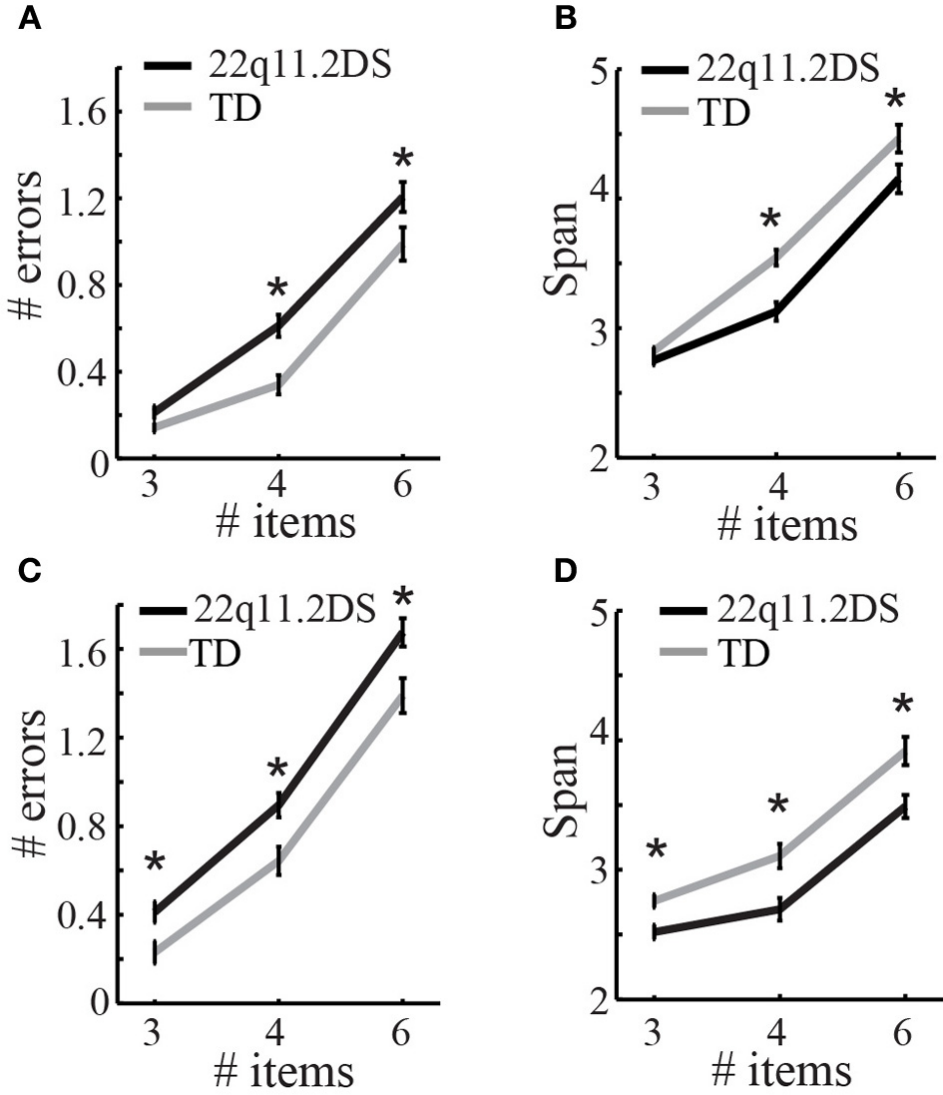
**Results of the working memory task, the self-ordered pointing test (SOPT)**. On the verbal version of the task, children with 22q11.2DS made more errors **(A)** and had a lower span **(B)** on the more difficult trials levels with 4 and 6 items to remember (^*^p < 0.05). On the non-verbal version of the SOPT, children with 22q11.2DS made significantly more errors **(C)** and had a lower span **(D)** across all levels of the non-verbal SOPT, when compared to TD children (^*^p < 0.05).

For the *verbal* version of the task, there was a significant group difference in span [*F*_(1, 113)_ = 11.40, *p* = 0.001], as well as a significant Group × Level interaction, such that children with 22q11.2DS performed more poorly than TD children at higher levels of difficulty relative to lower levels of difficulty [*F*_(2, 228)_ = 3.39, *p* = 0.04; Figure 5B]. Similarly, there was a significant group difference in number of errors [*F*_(1, 113)_ = 11.86, *p* = 0.0008; Figure 5A], though the Group × Level interaction here was not quite significant [*F*_(2, 228)_ = 2.69, *p* = 0.07]. See Supplementary Table 4 for group- and level-wise scores on each level, as well as the results of statistical tests for group differences in performance at each level.

For the *non-verbal* version of the task, there was a significant group difference in span [*F*_(1, 100)_ = 17.25, *p* = 0.0001], but no Group × Level interaction [*F*_(2, 202)_ = 1.092, *p* = 0.34; Figure 5D]. Similarly, there was a significant group difference in number of errors [*F*_(1, 100)_ = 15.08, *p* = 0.0002] and no Group × Level interaction [*F*_(2, 202)_ = 0.53, *p* = 0.59; Figure 5C]. The main difference between the results of the verbal vs. the non-verbal version of the test is that there was a Group × Level interaction in performance for the verbal, but not the non-verbal, version of the task. This is likely due to the fact that children with 22q11.2DS performed more poorly than TD children across all levels of the non-verbal version of the task, while they only performed comparably to TD children at easier levels of the verbal version of the task, and worse at more difficult levels. See Supplementary Table 4 for group- and level-wise scores on each level, as well as the results of statistical tests for group differences in performance at each level.

### Age and cognitive control

**Figure 6:**
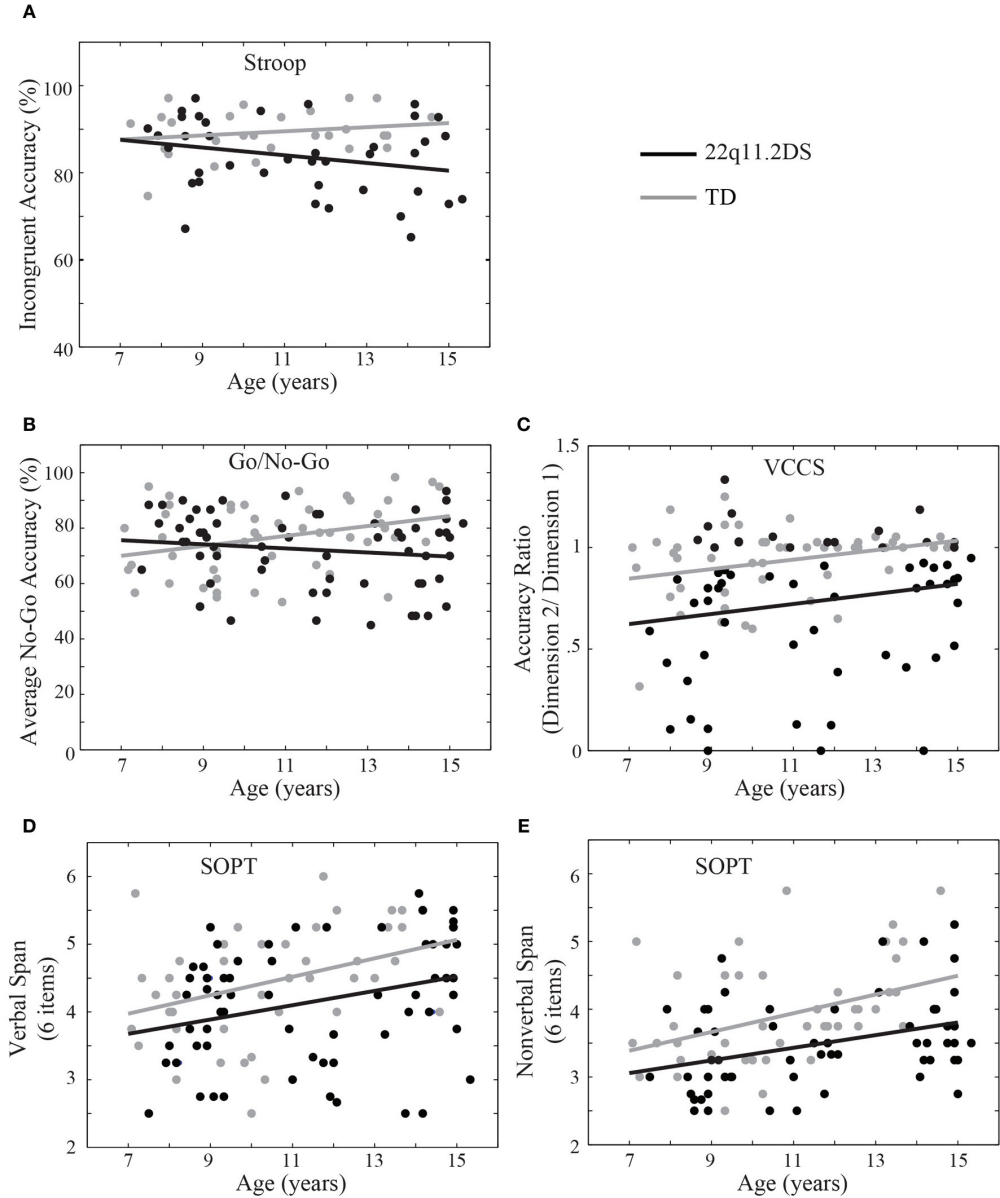
**Age-related associations with cognitive control component processes**. Age was not related to incongruent accuracy on the Stroop for either group **(A)**. On the Go/No-Go task, TD children demonstrated a significant age-related association with No-Go accuracy (*p* < 0.05) while children with 22q11.2DS did not **(B)**. Similarly, on the Visually-Cued Card Sort (VCCS), age was significantly associated with the ratio of Dimension 2 accuracy divided by that of Dimension 1 for TD children (*p* < 0.05) but not those with 22q11.2DS **(C)**. Age significantly correlated with span on the Self-Ordered Pointing Test (SOPT) for both TD and 22q11.2DS children on the verbal **(D)** and nonverbal **(E)** versions of the task (*p* < 0.05).

To examine the development of cognitive control in children with 22q11.2DS and TD children, age was included in the within-group regression models. For response inhibition, age was regressed on accuracy for the Stroop and Go/No-Go tasks. We found that age was not related to incongruent accuracy on the Stroop for either group [*F*_(1, 26)_ = 0.29, *p* = 0.59 and *F*_(1, 36)_ = 2.52, *p* = 0.12 for TD and 22q11.2DS, respectively; Figure 6A]. The scatterplot of this relationship (Figure 6A) illustrates that most TD children are performing at very high levels of accuracy on this task, while variance in performance appears to be increasing in older individuals with 22q11.2DS.

On the Go/No-Go task we found that TD children demonstrated a significant age-related association with No-Go accuracy [*F*_(1, 33)_ = 4.91, *p* = 0.03], such that older TD children performed better on the response inhibition task than younger TD children. By contrast, children with 22q11.2DS demonstrated no relationship of age with No-Go accuracy [*F*_(1, 46)_ = 0.53, *p* = 0.47; Figure 6B].

To examine the development of cognitive flexibility in the two groups, age was regressed on percent accuracy for each dimension of the VCCS (Dimensions 1 and 2 for 80 and 20% frequency, respectively), as well as the ratio of Dimension 2 accuracy divided by that of Dimension 1. TD children demonstrated significant associations of age with accuracy on both dimensions [*F*_(1, 47)_ = 6.53, *p* = 0.01 and *F*_(1, 47)_ = 11.81, *p* = 0.001 for Dimension 1 and 2, respectively], as did children with 22q11.2DS [*F*_(1, 59)_ = 3.51, *p* = 0.07 and *F*_(1, 59)_ = 5.38, *p* = 0.02 for Dimension 1 and 2, respectively]. With regard to the ratio of accuracy on Dimension 2 divided by that of Dimension 1, TD children again demonstrated a significant effect of age [*F*_(1, 47)_ = 4.48, *p* = 0.04], while children with 22q11.2DS did not [*F*_(1, 59)_ = 2.15, *p* = 0.15; Figure 6C].

To examine the development of working memory, age was regressed against span on the SOPT for each group. Here we found a significant age-related association with span for the verbal version of the SOPT for TD children [*F*_(1, 49)_ = 6.11, *p* = 0.02], as well as children with 22q11.2DS [*F*_(1, 61)_ = 6.24, *p* = 0.02; Figure 6D]. Similarly, both groups demonstrated significant age-related associations with span on the non-verbal version of the task [*F*_(1, 44)_ = 8.88, *p* = 0.005 and *F*_(1, 53)_ = 20.62, *p* < 0.0001 for TD and 22q11.2DS, respectively; Figure 6E]. See Supplementary Table 5 for a complete list of within-group statistical tests of age on cognitive control outcome measures.

### General intellectual ability and cognitive control

There was a significant group difference in full-scale IQ [FSIQ; mean[SD] = 74.8[12.0] for 22q11.2DS and 110.2[12.3] for TD; *F*_(1, 90)_ = 200.06, *p* < 0.0001]. To assess the relationship of general intellectual abilities to cognitive control function, FSIQ was included as a regressor against outcome measures on the cognitive control tasks. On the Stroop task, there were no effects of FSIQ on accuracy on incongruent trials within either of the groups [*F*_(1, 22)_ = 0.24, *p* = 0.63 and *F*_(1, 29)_ = 0.79, *p* = 0.38 for TD and 22q11.2DS, respectively]. Similarly, there were no effects of FSIQ on No-Go accuracy [*F*_(1, 32)_ = 0.51, *p* = 0.48 and *F*_(1, 47)_ = 0.88, *p* = 0.35 for TD and 22q11.2DS, respectively]. On the VCCS test of cognitive flexibility, FSIQ had a significant effect on the Dimension 2/Dimension 1 ratio for TD children [*F*_(1, 33)_ = 8.89, *p* = 0.005] but not children with 22q11.2DS [*F*_(1, 44)_ = 2.43, *p* = 0.13]. On the SOPT test of working memory, the only significant within-group relationship of FSIQ with span was that of non-verbal span with FSIQ in TD children [*F*_(1, 30)_ = 9.91, *p* = 0.004].

### COMT and cognitive control

**Figure 7:**
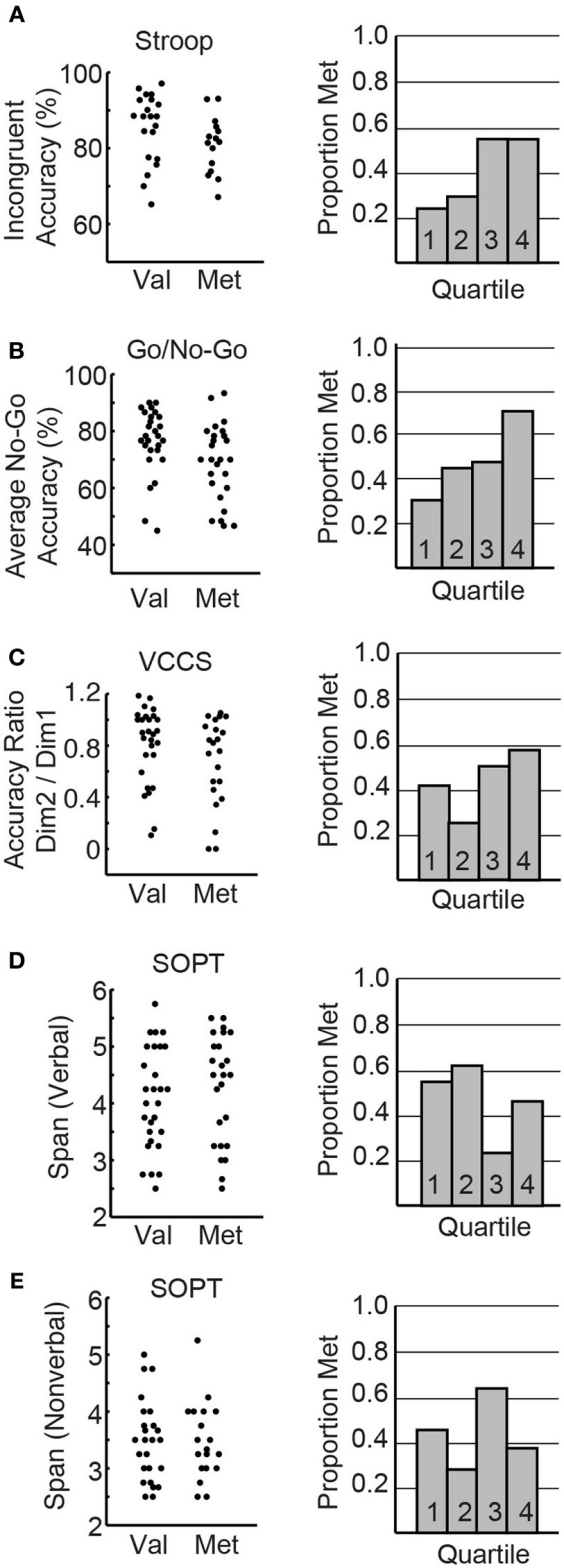
**COMT and cognitive control**. The left panel depicts the primary outcome measures for each task graphed as a function of COMT variant for the individual children with 22q11.2DS. **(A)** Incongruent accuracy on the Stroop task. **(B)** Average No-Go accuracy on the response inhibition Go/No-Go task. **(C)** Accuracy ratio (Dimension 2/Dimension 1) on the VCCS. **(D,E)** Verbal and non-verbal span, respectively, on the most difficult level of the self-ordered pointing test (6 items to remember). The right panels of the figure depict the proportion of individuals within each performance quartile of the particular task that had the Met variant of the COMT gene.

First, we wanted to visualize the relationship of COMT genotype to performance on the different cognitive control tasks, in order to assess whether or not specific COMT genotypes might account for some of the variance that is seen among individuals with 22q11.2DS. For each task, we graphed the primary outcome measure as a function of genotype for the children with 22q11.2DS (Figures 7A–E). Qualitatively, it appeared that on the response inhibition tasks, there were more individuals with the Met allele performing poorly relative to those with the Val allele. In order to quantify this observation, we split the participants into four groups based on their performance. The first group included those performing in the top quartile of the sample (highest performers), down to the fourth group that consisted of those performing in the fourth quartile of the sample (lowest performers). We then graphed the proportion of individuals within each quartile that had the Met allele (calculated by taking the number of participants within that sample that had the Met allele, divided by the total number of participants in that quartile; Figures 7A–E).

We also assessed potential COMT effects using regression models. COMT genotype was included as a regressor against outcome measures on the cognitive control tasks in children with 22q11.2DS. On the Stroop task, there was no effect of COMT on incongruent accuracy [*F*_(1, 32)_ = 1.40, *p* = 0.26]. By contrast, on the Go/No-Go task, COMT genotype had a significant effect on overall No-Go accuracy [*F*_(1, 50)_ = 4.54, *p* = 0.04], such that individuals with the Met allele had lower accuracy. There was no effect of COMT on the ratio of Dimension 2/Dimension 1 accuracy on the VCCS task [*F*_(1, 46)_ = 1.99, *p* = 0.16]. Similarly there was no effect of COMT on working memory span for either version of the SOPT task [*F*_(1, 49)_ = 0.89, *p* = 0.35 and *F*_(1, 41)_ = 0.40, *p* = 0.53 for verbal and non-verbal, respectively].

## Discussion

The present study was designed to investigate cognitive control and its age-related development in a cross-sectional sample of children with 22q11.2DS. As expected based on the literature (Sobin et al., 2005; Lewandowski et al., 2007; Campbell et al., 2010), when compared to TD controls, children with 22q11.2DS were impaired on all three cognitive control foundational processes: response inhibition, cognitive flexibility, and working memory. The advantage of this study is that it enabled us to examine individual performance patterns across a battery of tasks within the same sample of participants, thus identifying relative strengths and weaknesses in cognitive control component processes that might generate hypotheses about specific mechanisms underpinning cognitive control impairments. Importantly, by examining these processes across an age range of children with 22q11.2DS and TD controls, we were able to conduct a cross-sectional analysis of developmental trajectories.

As expected, TD children demonstrated a significant effect of age on most cognitive control component processes, such that older children had better performance relative to their younger counterparts. The only measure on which TD children did not demonstrate an age-related association was that of Stroop incongruent accuracy, likely due to nearly ceiling effects across all ages (Figure 6A). By contrast, children with 22q11.2DS demonstrated no age-related associations within our 7–14 year age range on four of the tasks, including Stroop, Go/No-Go, and VCCS. Analysis of individual performance patterns on the response inhibition tasks (Stroop and Go/No-Go) suggested that some of the older children with 22q11.2DS performed similarly to TD children while others performed much worse. Thus, an atypical developmental trajectory of response inhibition in this population was due to increased variability of performance in older individuals with the disorder. The inter-individual variability seen in older individuals with 22q11.2DS may contain great value with respect to identifying individuals whose inhibitory function is developing atypically relative to their peers, thus providing insight into mechanisms that might be underpinning variability within the group. Distinguishing measures such as these, especially those that have been linked to cognitive dysfunction in schizophrenia, are valuable targets to explore for better understanding individuals that are at greater risk for psychopathology. It will be important to explore these developmental patterns longitudinally in future studies.

This same sample of participants also demonstrated an atypical age-related association with cognitive flexibility. While general card sorting ability on the task had a similar age-related association in the two groups, the ability to sort by the less dominant dimension was not only impaired in children with 22q11.2DS, but also did not show the typical effect of improving with age that was apparent in the TD participants. Similar to the response inhibition results, this overall group effect was due to increased variability of performance in older individuals with 22q11.2DS, with some performing well and others highly impaired.

In contrast to the atypical age effects seen in response inhibition and cognitive flexibility, the children with 22q11.2DS demonstrated a typical relationship of age with span on the working memory task. This preliminary cross-sectional sample suggests that, despite an overall impairment in performance on this task, the development of this component of cognitive control in 22q11.2DS might be more typical than the others. One possible implication here is that the neural circuitry supporting working memory is developing and becoming more efficient at a rate similar to TD individuals. Alternatively, it is possible that compensatory mechanisms support improved performance on the working memory task in this age range of individuals with 22q11.2DS.

It is important to think about these results in the context of a framework for cognitive control, while remembering that the distinct sub-components are neither pure nor perfect with respect to their distinctions, as well as the tasks proposed to measure them. As described by Miyake et al. (2000), this system is likely composed of foundational cognitive control components that are both distinct and interrelated. Additionally, with respect to the tasks designed to measure these components, there will surely be overlap in the functions required for completing each task. The Go/No-Go task requires inhibitory control in order to inhibit a pre-potent response to press the button on frequently occurring “Go” trials. This task also requires some working memory in order to remember which stimuli are indicative of a Go trial and which stimuli represent a No-Go trial. The VCCS requires participants to follow specific rules and to be cognitively flexible in order to respond appropriately to the given rules that change according to certain criteria. Working memory is necessary to remember the current rules at hand. Additionally, inhibitory control is required in order to inhibit the inclination to respond according to the predominant sorting dimension. Finally, the SOPT requires participants to hold a number of items in working memory, while also comparing responses that have already been made with those that will be made in the future (self-monitoring). This type of behavior also requires some degree of planning and organization. While it is important to recognize that the neurocognitive tasks here might be multi-componential to some degree, their unique emphasis on specific cognitive processes is important to recognize, and the overlapping nature provides an opportunity to compare performance on different components with respect to their primary and overlapping functionalities.

The results of the current study suggest a specific aberration in 22q11.2DS in the development of networks mediating response inhibition and cognitive flexibility. One unifying feature of the response inhibition and cognitive flexibility tasks that distinguishes them from the working memory task is that the former two both require the ability to inhibit a pre-potent response. Given the component of inhibitory control that is required to sort by the less dominant dimension on the VCCS, it is unclear the extent to which difficulties with response inhibition might underlie performance on this task of cognitive flexibility. One approach to examining the specific and interrelated nature of cognitive control component processes could be through latent variable analyses and computational modeling (Miyake et al., 2000; Friedman and Miyake, 2004; Miyake and Friedman, 2012). These would be important studies in the future for better understanding the most specific nature of cognitive control impairments in 22q11.2DS.

Cognitive control impairments are exceedingly common in other neurodevelopmental and psychiatric disorders. In order to examine the specificity of cognitive control impairments in 22q11.2DS, and whether or not group differences in global cognitive functioning (non-specific to 22q11.2DS) were driving the results, FSIQ was included as a regressor against outcome measures on the cognitive control tasks. We found that FSIQ was not related to task performance in the children with 22q11.2DS, thus suggesting that the observed impairments in cognitive control were not being driven by global cognitive functioning, as measured by FSIQ. It is important to mention, however, that FSIQ alone is not necessarily a comprehensive measure of global cognitive functioning, and that future work will be needed in order to more directly examine the relationship of general intelligence to cognitive control in 22q11.2DS. For example, it would be important to examine the relationship of cognitive control impairments in 22q11.2DS to fluid intelligence, which is believed to reflect abstract reasoning and problem solving skills, a functionality that is impaired after lesions of the frontal lobe (Duncan et al., 1995). It has been demonstrated that, in a population of patients with frontal lesions, there were no specific deficits related to cognitive control once fluid intelligence was taken into account (Roca et al., 2010). It is likely, however, that cognitive control impairments are not fully explained by fluid intelligence. Other evidence suggests that the different cognitive control component processes (inhibition, cognitive flexibility, and working memory) are differentially related to fluid intelligence (Friedman et al., 2006). The specificity of these impairments and their relationship to fluid intelligence remain to be parsed in developmental disorders, and this will be an important question to pursue in children with 22q11.2DS.

Another important issue related to understanding the specificity of cognitive impairments and their developmental courses in 22q11.2DS is the selection of an appropriate comparison population. In the present study there are limitations associated with matching developmentally delayed individuals with age-matched typical controls. However, matching by mental age or by cognitive ability would introduce additional variables and confounds. More specifically, a comparison group matched by cognitive ability would involve a highly heterogeneous sample of participants with many different etiologies. At present we felt that, even despite the limitations, it was important to observe performance in 22q11.2DS relative to age-matched TD controls. For one, it affords the opportunity to draw comparisons from a representative control sample as opposed to a heterogenous sample (Dennis et al., 2009). Importantly, this design allows us to estimate the magnitude of impairment in 22q11.2DS relative to age-matched TD controls, thus establishing a baseline that can be used as a reference in the future for studies of intervention. Potential timelines for cognitive control development have been described in TD children (Huizinga et al., 2006; Best and Miller, 2010). In the current study we wanted to assess how the developmental trajectories of cognitive control in 22q11.2DS might compare to the standard in TD individuals, given that atypical neurodevelopmental trajectories are exceedingly common in childhood psychiatric disorders (Shaw et al., 2010). For this comparison, it is important to include TD participants in order to first replicate the existing data and show that the current tasks are validly reproducing well-established developmental time courses in the TD group. Subsequently, we can accurately assess the differences in developmental trajectories between TD and 22q11.2DS, as well as within children with 22q11.2DS.

In addition to cognitive analysis, the current study also examined COMT genotype as a function of performance on the different cognitive control tasks, in order to assess whether or not specific COMT genotypes might account for some of the variance that is seen among individuals with 22q11.2DS. Interestingly, it appeared that the children with 22q11.2DS who were hemizygous for the Met variant of the COMT gene performed more poorly on the tasks of response inhibition relative to their peers with 22q11.2DS who were hemizygous for the Val allele. Though this relationship was only statistically significant when assessing performance on the Go/No-Go task, it appeared that there was a trend toward this relationship in the other tasks with inhibitory requirements, including the Stroop and VCCS (see Figures 7A–C). By contrast, this was not the case for the SOPT task of working memory (Figures 7D,E). This is an interesting dissociation, given that previous studies have suggested that inhibitory tasks are more dependent on DA than the SOPT (Diamond et al., 1997; Collins et al., 1998). These results indicate that participants with 22q11.2DS who were hemizygous for the Met allele tended to perform worse on the tasks that have previously been suggested to be DA-dependent. According to the model that the effect of DA on cognition follows an inverse U pattern, with an optimal range of DA involving not too much or too little of the neurotransmitter, it is reasonable to assume that children with 22q11.2DS and the Met allele for COMT are at a disadvantage relative to those with Val. After all, since children with 22q11.2DS are already hemizygous for COMT, it is likely that they have less prefrontal COMT activity and higher levels of DA. Thus, hemizygosity for Val, the variant with greater catalytic activity, would be more advantageous for maintaining a position closer to the optimal peak of prefrontal DA as it relates to higher-level cognitive processes. This hypothesis will have to be tested further in the future.

It is not surprising that discrepancies in COMT effects in 22q11.2DS are often reported in the literature. After all, the effects of a single gene are not likely to be very powerful, and impact might also vary as a function of other factors such as age, gender (Kates et al., 2006), or other genetic variants (Vorstman et al., 2009). In addition to the issue of power, another reason for the limited and inconclusive reports on the relationship of genetic variants to cognitive function is that genetics are likely influenced by environmental factors. Two noteworthy factors are stress and anxiety. While the genetics of 22q11.2DS predispose individuals to susceptibility for greater stress and anxiety, it is possible that mechanisms for coping and reducing these influences will contribute to better adaptive function and thus better long-term outcomes (Beaton and Simon, 2011; Angkustsiri et al., 2012).

It is reasonable to assume that the observed cognitive control impairments in 22q11.2DS are in some way mediated by the genetics of the disorder, and are subserved by underlying impairments in neural architecture that supports these cognitive processes. Cognitive control is largely mediated by activity within the prefrontal cortex (PFC) and reciprocal connections between the PFC and subcortical networks. In humans and monkeys, damage to the dorsolateral PFC (dlPFC) impairs performance on the Go/No-Go task (Iversen and Mishkin, 1970), the VCCS (Passingham, 1972; Dias et al., 1996), and the SOPT (Petrides and Milner, 1982; Petrides, 1991). Additionally, neuroimaging studies have demonstrated that the dlPFC is more active during each respective cognitive task when compared to a control task (Petrides et al., 1993; Berman et al., 1995; Casey et al., 1997).

Frontally-mediated regulation of cognitive control is often modulated by subcortical circuitry. One of the key components of this system is frontostriatal circuitry, which involves neuronal loops connecting the PFC, thalamus, and basal ganglia. The basal ganglia consist of interconnected subcortical nuclei that receive major input from the cerebral cortex and thalamus, and then connect back to the cerebral cortex via the thalamus (Alexander et al., 1986). There is some evidence that these circuits are atypical in 22q11.2DS. Structural imaging studies have demonstrated GM reduction and dysfunction in 22q11.2DS (Shashi et al., 2010), as well as alterations in midline cortical thickness and gyrification patterns (Bearden et al., 2009). There is also evidence for atypical basal ganglia structure in 22q11.2DS (Sugama et al., 2000; Eliez et al., 2002), as well as atypical structural connectivity within frontal networks (Simon et al., 2008). Functional imaging studies have also demonstrated irregularities in these networks in children with 22q11.2DS when compared to TD children, including atypical parietal activity during a Go/No-Go task (Gothelf et al., 2007a), as well as hypoactivation of dorsolateral PFC during performance on a working memory task (Kates et al., 2007).

With respect to the structural and functional developmental of cognitive control neural networks in 22q11.2DS, evidence suggests that the developmental trajectory of cortical gyrification is atypical in children with 22q11.2DS relative to TD children in this age range (6–15 years) (Srivastava et al., 2011). The specific nature and timing of these trajectories are still unclear, however, and to date there have only been a few longitudinal studies of developmental trajectories of brain structure in 22q11.2DS (Gothelf et al., 2007b; Schaer et al., 2009; Kates et al., 2011; Kunwar et al., 2012). While these studies indicated neuroanatomical differences in frontal and parietal regions in children and adolescents with 22q11.2DS relative to TD individuals, evidence for atypical development trajectories was inconsistent. Larger samples of longitudinal studies during this critical developmental time period will be important for more directly examining the development of brain and behavior relationships responsible for cognitive control in 22q11.2DS.

A better understanding of genes, brain, behavior, and external modulatory components of cognitive control in 22q11.2DS is most relevant given the high risk of schizophrenia in this population. Approximately 25% of individuals with 22q11.2DS will develop schizophrenia by adulthood (Murphy et al., 1999), rendering it the highest genetic risk factor for the disorder after having two parents or a twin sibling with schizophrenia. There is evidence for attenuated cognitive control impairments among first-degree relatives of individuals with schizophrenia, suggesting that these deficits might be part of an endophenotype related to genetic susceptibility for the disorder (Snitz et al., 2006). Thus, the results of the current study pose interesting questions as to whether aberrant response inhibition might be part of an endophenotype for schizophrenia risk in 22q11.2DS, and if so might the lower-performing older individuals with 22q11.2DS be the individuals at greatest risk for conversion? These are questions that will be explored in the future via longitudinal analyses and correlations with measures of psychosis. In this manner, we will be able to directly examine the potential for these kinds of tasks as non-invasive diagnostic measures for risk probability, or as evaluative tools for the efficacy of targeted interventions (Carter and Barch, 2007).

In sum, these results point toward a specific aberration in the development of systems mediating response inhibition in a sub-set of the children with 22q11.2DS, at a critical age when these individuals are at significant risk for developing schizophrenia. Though the present study was cross-sectional in design, it provides a valuable starting point for longitudinal analyses. In the future it will be important to directly examine developmental trajectories that integrate genetic, physiological, neurocognitive, and clinical psychosis measures in order to obtain a most comprehensive picture of modulatory factors pertaining to the development of cognitive control, as well as clinical and adaptive outcomes.

### Conflict of interest statement

The authors declare that the research was conducted in the absence of any commercial or financial relationships that could be construed as a potential conflict of interest.
