# Supplementary material for: The development of cognitive control in children with chromosome 22q11.2 deletion syndrome
Source: Front Psychol. 2014 Jun 10;5:566. doi: 10.3389/fpsyg.2014.00566 (PMC4050531; doi:10.3389/fpsyg.2014.00566)
Supplement: Supplementary file 1 [file DataSheet1.DOCX]

***Supplementary Material***

**The Development of Cognitive Control in Children with Chromosome 22q11.2 Deletion Syndrome**

**Heather M. Shapiro^1§^, Flora Tassone^1,2^, Nimrah Choudhary^2^, and Tony J. Simon^1^**

^1^ MIND Institute and Department of Psychiatry and Behavioral Sciences, University of California at Davis, Sacramento, CA, USA

^2^ Department of Biochemistry and Molecular Medicine, University of California at Davis, Sacramento, CA, USA

^§^ Address correspondence to:

Heather M. Shapiro, PhD

MIND Institute, 2825 50^th^ Street, Room 1357

Sacramento, CA 95817

Email: hmshapiro@ucdavis.edu

Tel: 916-703-0407

Fax: 916-703-0244

**Supplementary Table 1**

Response inhibition outcome measures from the Stroop task

|  |  | **22q11.2DS**  **Mean (SD)** | **TD**  **Mean (SD)** | **Between-group test___**  ***F β*±SE *p*** | | |
| --- | --- | --- | --- | --- | --- | --- |
| ***Accuracy (%)*** | **cC** | 94.0 (3.9) | 96.7 (3.4) | 8.96 | -2.73±.90 | .0004* |
| **by Trial Type:** | **iC** | 93.3 (6.2) | 96.3 (4.3) | 4.89 | -2.98±1.35 | .03* |
|  | **cI** | 83.8 (9.1) | 88.4 (5.4) | 8.53 | -5.65±1.91 | .005* |
|  | **iI** | 85.8 (11.0) | 92.1 (8.3) | 6.66 | -6.39±2.44 | .01* |
| ***RT (ms)*** | **cC** | 765.8 (166.0) | 726.4 (162.6) | .94 | 40.09±40.67 | .34 |
| **by Trial Type:** | **iC** | 834.2 (192.5) | 762.1 (202.4) | 2.20 | 72.22±48.65 | .14 |
|  | **cI** | 928.5 (221.0) | 906.1 (292.0) | .13 | 21.80±62.69 | .72 |
|  | **iI** | 961.2 (243.2) | 928.1 (299.4) | .25 | 33.27±66.42 | .62 |
| ***RT Difference (ms)***  **iC – cC** |  | 68.3 (59.8) | 35.7 (57.8) | 5.06 | 32.13±14.52 | .03* |

*Note*. Accuracy and response time (RT) are listed for each group respectively. Mixed model regression analyses were used to test group differences, and above you will see the test statistics for these models, as well as the associated coefficients and standard errors (ß ±SE). These coefficients represent between-group differences in the outcome measures listed above. For example, children with 22q11.2DS have an accuracy that is 2.73 points lower than TD on cC trials, on average.

**Supplementary** **Table 2**

Response inhibition outcome measures from the Go/No-Go task

|  |  | **22q11.2DS**  **Mean (SD)** | **TD**  **Mean (SD)** | **Between-group test___**  ***F β*±SE *p*** | | |
| --- | --- | --- | --- | --- | --- | --- |
| ***No-Go Accuracy (%)*** | **1** | 72.2 (15.0) | 70.5 (18.7) | .26 | 1.62±3.29 | .61 |
| **# prior Go trials:** | **3** | 72.6 (15.3) | 77.7 (14.7) | 3.17 | -5.35±2.86 | .08 |
|  | **5** | 72.0 (17.6) | 81.7 (14.0) | 9.95 | -9.56±3.09 | .002* |
| ***Go RT (ms)*** | **1** | 406.3 (81.1) | 407.9 (63.3) | .01 | -1.04±14.14 | .91 |
|  | **2** | 440.5 (89.2) | 417.8 (74.8) | 2.05 | 21.82±15.92 | .16 |
| **Trial type** | **3** | 444.5 (83.7) | 431.9 (75.7) | .68 | 11.05±15.29 | .41 |
| **following No-Go:** | **4** | 458.3 (87.6) | 454.0 (78.8) | .07 | 2.94±16.00 | .79 |
|  | **5** | 444.3 (90.5) | 447.6 (80.2) | .04 | -5.20±16.37 | .84 |

*Note*. Accuracy and response time (RT) are listed for each group respectively. Mixed model regression analyses were used to test group differences, and above you will see the test statistics for these models, as well as the associated coefficients and standard errors (ß ±SE). These coefficients represent between-group differences in the outcome measures listed above. For example, children with 22q11.2DS have an accuracy that is 9.56 points lower than TD on No-Go trials that follow 5 preceding Go trials, on average.

**Supplementary** **Table 3**

Cognitive flexibility outcome measures from the Visually-Cued Card Sort (VCCS)

|  |  | **22q11.2DS**  **Mean (SD)** | **TD**  **Mean (SD)** | **Between-group test___**  ***F β*±SE *p*** | | |
| --- | --- | --- | --- | --- | --- | --- |
| ***Accuracy (%):*** | **Dim1** | 86.13 (12.30) | 94.65 (7.78) | 18.11 | -8.39±2.01 | <.0001* |
|  | **Dim2** | 63.71 (29.99) | 88.18 (17.10) | 26.22 | -24.24±4.79 | <.0001* |
| ***Accuracy Ratio: Dim2/Dim1*** |  | .74 (.33) | .93 (.17) | 14.45 | -.20±.05 | .0002* |

*Note*. Accuracy and the accuracy ratio of dimension 2 divided by dimension 1 (Dim2/Dim1) are listed for each group respectively. Mixed model regression analyses were used to test group differences, and above you will see the test statistics for these models, as well as the associated coefficients and standard errors (ß ±SE). These coefficients represent between-group differences in the outcome measures listed above. For example, children with 22q11.2DS have accuracy on dimension 1 (Dim1) that is 18.11 points lower than TD, on average.

**Supplementary** **Table 4**

Working memory outcome measures from the Self-Ordered Pointing Test (SOPT)

|  |  | **22q11.2DS**  **Mean (SD)** | **TD**  **Mean (SD)** | **Between-group test___**  ***F β*±SE *p*** | | |
| --- | --- | --- | --- | --- | --- | --- |
| ***Verbal Span*** | **3** | 2.75 (0.31) | 2.82 (0.23) | 1.88 | -.07±.05 | .17 |
| **Trial Type:** | **4** | 3.13 (0.58) | 3.54 (0.44) | 18.09 | -.42±.10 | <.0001* |
|  | **6** | 4.15 (0.90) | 4.46 (0.78) | 3.91 | -.29±.16 | .05* |
| ***Verbal Errors*** | **3** | 0.21 (0.25) | 0.14 (0.16) | 2.90 | .07±.04 | .09 |
| **Trial Type:** | **4** | 0.61 (0.41) | 0.34 (0.32) | 15.53 | .27±.07 | .0001* |
|  | **6** | 1.21 (0.55) | 0.99 (0.55) | 4.61 | .20±.10 | .03* |
| ***Nonverbal Span*** | **3** | 2.52 (0.42) | 2.76 (0.35) | 9.85 | -.23±.08 | .002* |
| **Trial Type:** | **4** | 2.69 (0.66) | 3.11 (0.65) | 10.05 | -.41±.13 | .002* |
|  | **6** | 3.49 (0.67) | 3.92 (0.75) | 9.28 | -.42±.14 | .003* |
| ***Nonverbal Errors*** | **3** | 0.41 (0.36) | 0.23 (0.35) | 7.00 | .17±.07 | .009* |
| **Trial Type:** | **4** | 0.90 (0.41) | 0.64 (0.44) | 8.99 | .26±.08 | .003* |
|  | **6** | 1.68 (0.48) | 1.39 (0.54) | 8.09 | .29±.10 | .005* |

*Note*. Accuracy and the accuracy ratio of dimension 2 divided by dimension 1 (Dim2/Dim1) are listed for each group respectively. Mixed model regression analyses were used to test group differences, and above you will see the test statistics for these models, as well as the associated coefficients and standard errors (ß ±SE). These coefficients represent between-group differences in the outcome measures listed above. For example, children with 22q11.2DS have accuracy on dimension 1 (Dim1) that is 18.11 points lower than TD, on average.

**Supplementary** **Table 5**

Age-related associations with cognitive control measures

|  | **_____TD_____** | |  | **__22q11.2DS__** | |
| --- | --- | --- | --- | --- | --- |
|  | **F** | ***p*** |  | **F** | ***p*** |
| ***Stroop: Incongruent Accuracy * Age*** | .29 | .59 |  | 2.52 | .12 |
| ***Go/No-Go: No-Go Accuracy * Age*** | 4.98 | .03* |  | 1.31 | .26 |
| ***VCCS: Accuracy Ratio (Dim2/Dim1 * Age*** | 4.48 | .04* |  | 2.15 | .15 |
| ***SOPT (verbal): Span * Age*** | 6.11 | .02* |  | 6.24 | .02* |
| ***SOPT (nonverbal): Span * Age*** | 8.88 | .005* |  | 20.62 | <.0001* |
